# Radiological Mapping of Post-Disaster Nuclear Environments Using Fixed-Wing Unmanned Aerial Systems: A Study From Chornobyl

**DOI:** 10.3389/frobt.2019.00149

**Published:** 2020-01-17

**Authors:** Dean T. Connor, Kieran Wood, Peter G. Martin, Sevda Goren, David Megson-Smith, Yannick Verbelen, Igor Chyzhevskyi, Serhii Kirieiev, Nick T. Smith, Tom Richardson, Thomas B. Scott

**Affiliations:** ^1^Interface Analysis Centre, University of Bristol, Bristol, United Kingdom; ^2^Aerospace Engineering, University of Bristol, Bristol, United Kingdom; ^3^South West Nuclear Hub, University of Bristol, Bristol, United Kingdom; ^4^SSE Ecocentre, Ministry of Ecology, Chornobyl, Ukraine; ^5^National Nuclear Laboratory, Workington, United Kingdom

**Keywords:** radiation, Chornobyl, UAS (unmanned aircraft system), fixed-wing aerial surveys, post-disaster, cesium, nuclear, drones (UAV)

## Abstract

In the immediate aftermath following a large-scale release of radioactive material into the environment, it is necessary to determine the spatial distribution of radioactivity quickly. At present, this is conducted by utilizing manned aircraft equipped with large-volume radiation detection systems. Whilst these are capable of mapping large areas quickly, they suffer from a low spatial resolution due to the operating altitude of the aircraft. They are also expensive to deploy and their manned nature means that the operators are still at risk of exposure to potentially harmful ionizing radiation. Previous studies have identified the feasibility of utilizing unmanned aerial systems (UASs) in monitoring radiation in post-disaster environments. However, the majority of these systems suffer from a limited range or are too heavy to be easily integrated into regulatory restrictions that exist on the deployment of UASs worldwide. This study presents a new radiation mapping UAS based on a lightweight (8 kg) fixed-wing unmanned aircraft and tests its suitability to mapping post-disaster radiation in the Chornobyl Exclusion Zone (CEZ). The system is capable of continuous flight for more than 1 h and can resolve small scale changes in dose-rate in high resolution (sub-20 m). It is envisaged that with some minor development, these systems could be utilized to map large areas of hazardous land without exposing a single operator to a harmful dose of ionizing radiation.

## 1. Introduction

The large-scale release of radionuclides from the Chornobyl Nuclear Power Plant (ChNPP) remains the most significant nuclear accident in the history of civil nuclear power generation. During the 10 days of emissions from Reactor 4, approximately 11,780 PBq of radioactive material was released into the environment, including 1,700 PBq of 131I and 85 PBq of ^137^Cs (Gudiksen et al., 1988; Cort et al., 1998; Smith and Beresford, 2005, p. 12). While the accident had far reaching environmental implications for a large area of Northern Europe, the area worst affected by the accident covers approximately 4,730 km^2^ across modern day Ukraine (2,600 km^2^) and Belarus (2,130 km^2^). The area within Ukraine defines the Chornobyl Exclusion Zone (CEZ), which is an access controlled region, established in May 1986, designed to mitigate dose exposure to the public. Restrictions on access are still in place to this day, although tourism permits are currently available as part of official guided tours.

In the decades since the accident, there have been significant advancements in remote/automated characterization technologies that were not available to the responding forces in April 1986. Examples of these technologies include radiation-hard robotics systems, stand-off characterization systems and nuclear-focused unmanned aerial systems (UAS). In particular, UAS with radiation mapping capabilities have been shown on a number of occasions to provide excellent results when it comes to mapping radiation within post-disaster environments.

There are a number of formats of UASs that have been used to map radiation within the environment, these include helicopter-style systems (Towler et al., 2012; Furutani et al., 2013; Sanada and Torii, 2014), multi-rotor systems (MacFarlane et al., 2014; Martin et al., 2015, 2016; Burtniak et al., 2018; Connor et al., 2018a,b) and fixed-wing systems (Kurvinen et al., 2005; Pöllänen et al., 2009). Operating within the area affected by the Fukushima nuclear incident in 2011, Sanada and Torii (2014) demonstrated that UASs are capable of greatly improving the resolution of airborne radiation maps vs. the traditional method of using manned aircraft systems (MAS). Using a Yamaha-RMAX helicopter as a transport platform for a custom radiation mapping payload, several plumes of radioactivity were revealed that were not identifiable within the original manned aircraft survey. This result confirmed that UAS can offer greater detail within airborne radiometric surveys, albeit at the expense of absolute spatial coverage due to the increased time it takes to survey a comparable area.

As well as the heavier systems, like the RMAX presented in Sanada and Torii (2014) (94 kg), smaller UAS systems have been effectively demonstrated for a range of radiological mapping purposes. These include post-disaster environments (Martin et al., 2016; Burtniak et al., 2018; Connor et al., 2018a,b), indoor applications (Boudergui et al., 2011) and mapping naturally occurring radioactive material (NORM) (Martin et al., 2015; Šálek et al., 2018). A full review of UAS radiation mapping is provided within Connor et al. (2016). More recently, a lightweight, multi-rotor UAS was used to map radiation within the “Red Forest” region of the CEZ in especially high resolution (5 m pixel^−1^). The system used within this study showed the capabilities of UASs to operate within even some of the most extreme radiological environments on earth with great effect. The ability of lightweight UASs to fly significantly lower than manned aircraft means that they can achieve improved spatial resolutions and sensitivities despite carrying much lighter (smaller-volume) payloads.

Terrestrial radiological measurements acquired from the air require a series of processing steps to convert the collected information into a true estimate of the activity of radioactive material present in the surveyed environment. Over approximately seven decades of operational experience of using manned aircraft, a defined series of processing steps have been established to accurately perform the correction of the raw measurements to a ground activity or dose-rate. This process is termed the “Spectral Windows” method and involves the segregation of the recorded gamma spectrum into a number of discrete energy windows that correspond to particular isotopes. The method is outlined in detail within Minty (1997) and can be used to map a range of radionuclides within the environment. However, these processing algorithms are optimized for large-volume (16–64 L) detectors that span gamma-ray energy ranges from 0 to 3 MeV, which are commonly used within manned aircraft surveys (International Atomic Energy Agency, 1991; Erdi-Krausz et al., 2003). The sensor packages used on unmanned aerial systems (UASs) are limited in terms of their volume (and hence detection efficiency) due to weight limits imposed by both physical and regulatory controls. This, in turn, limits certain key detection parameters that do not easily permit the use of all the steps within the standard processing algorithm encouraged by the IAEA. As increasing the payload capacity of UASs or reducing regulatory control is not often realistic or possible, alternative processing methods are required to overcome these hardware issues.

The major step within the workflow that small-volume systems struggle to overcome is in defining spectral stripping coefficients for removing scattered photons from erroneously appearing within lower energy spectral windows. This process occurs when an incident photon deposits only a portion of its energy to the detector medium before escaping the active volume without depositing further energy (Minty, 1997; Knoll, 2010). The occurrence is more pronounced in small-volume detectors due to the reduced interaction volume and causes the energy of the incident photon to be underestimated. The presence of high-energy emitters within the survey environment can therefore cause the intensity of lower-energy emitters to be overestimated. By using data acquired from well-characterized, infinite-yield sources (typically doped concrete pads) the magnitude of scattered photons falling within lower-energy windows can be determined through the definition of a series of simultaneous equations. These allow for the contribution to any energy window from one that exists at a higher energy to be removed, leaving only the true intensity behind. To correct for this, a signal within higher energy windows is required. This can be a problem for small-volume systems, as with limited detection volumes comes limited energy ranges. The contributions from abundant naturally occurring radioactive radionuclides (principally ^238^U/^235^U, ^232^Th, and ^40^K) need to be determined regardless of the primary target of the mapping procedure either for direct observation or for correction purposes. However, the characteristic spectral windows span energies in excess of 1.3 Mev (Table 1), which would require relatively large counting times or very intense sources to be able to accurately detect using small-volume detectors under aerial survey conditions. Therefore an alternative method for conducting this correction is required.

**Table 1:** Recommended spectral windows for radioelement mapping using manned aircraft.

**Window**	**Nuclide**	**Energy range (MeV)**
Total count	_	0.400–2.810
Cesium	^137^Cs (0.662 MeV)	0.618–0.705
Potassium	^40^K (1.460 MeV)	1.370–1.570
Uranium	^214^Bi (1.765 MeV)	1.660–1.860
Thorium	^208^Tl (2.614 MeV)	2.410–2.810

*Modified from International Atomic Energy Agency (1991) and Erdi-Krausz et al. (2003)*.

A further issue with utilizing small UASs into radiation mapping procedures is the limited battery life of the systems (Connor et al., 2016). This means that the absolute range of the aircraft is relatively low and therefore the potential area that can be covered in a single survey is small. This is highlighted in the research outlined above, as the investigations are limited to either relatively small sites, or present large data collection periods for more extensive areas. Increasing the range of radiation mapping UASs is therefore a necessary advancement for utilizing these platforms to monitor radiation over larger areas. At present, manned-aircraft are the most utilized method of radiologically mapping large areas of land. However, there are a number of problems with using this method over utilizing UASs. Firstly, manned aircraft cannot operate at very low altitudes in the same way that UASs can due to regulatory restrictions. This dramatically reduces the spatial of the radiometric data collected by the platform (Connor et al., 2016). Furthermore, manned aicraft surveys are inherently more expensive to conduct than UAS surveys and still require the exposure of the pilot/crew to potentially harmful ionizing radiation. One of the methods to overcome the limitations presented by both multi-rotor UASs and manned aircraft is to utilize fixed-wing vehicles as the transport platform. Whilst designs and implementations of radiation mapping systems using these aircraft have been presented within the literature (Kurvinen et al., 2005; Pöllänen et al., 2009), they have never been deployed to map terrestrial radiation in non-controlled environments.

A potential reason for the lack of real-world implementations is that, from an operational perspective, using a fixed-wing UAS is more complicated than using an equivalent system incorporating a multi-rotor vehicle. This arises from the physical differences in the way each platform achieves flight and maneuvers in the air. Multi-rotor systems fundamentally act like helicopters with the ability to statically hover, maneuver along complex flight paths and take-off/land in confined spaces. Advances in autopilot technology effectively isolates the pilot from the vehicle behavior with commands in the form of simplified orthogonal motions. Conversely, fixed-wing systems require constant motion to stay aloft and typically require much larger open areas for take-off/landing. The pilot also needs to be more skilled and guide the aircraft using combined roll and pitch motions.

**Figure 1:**
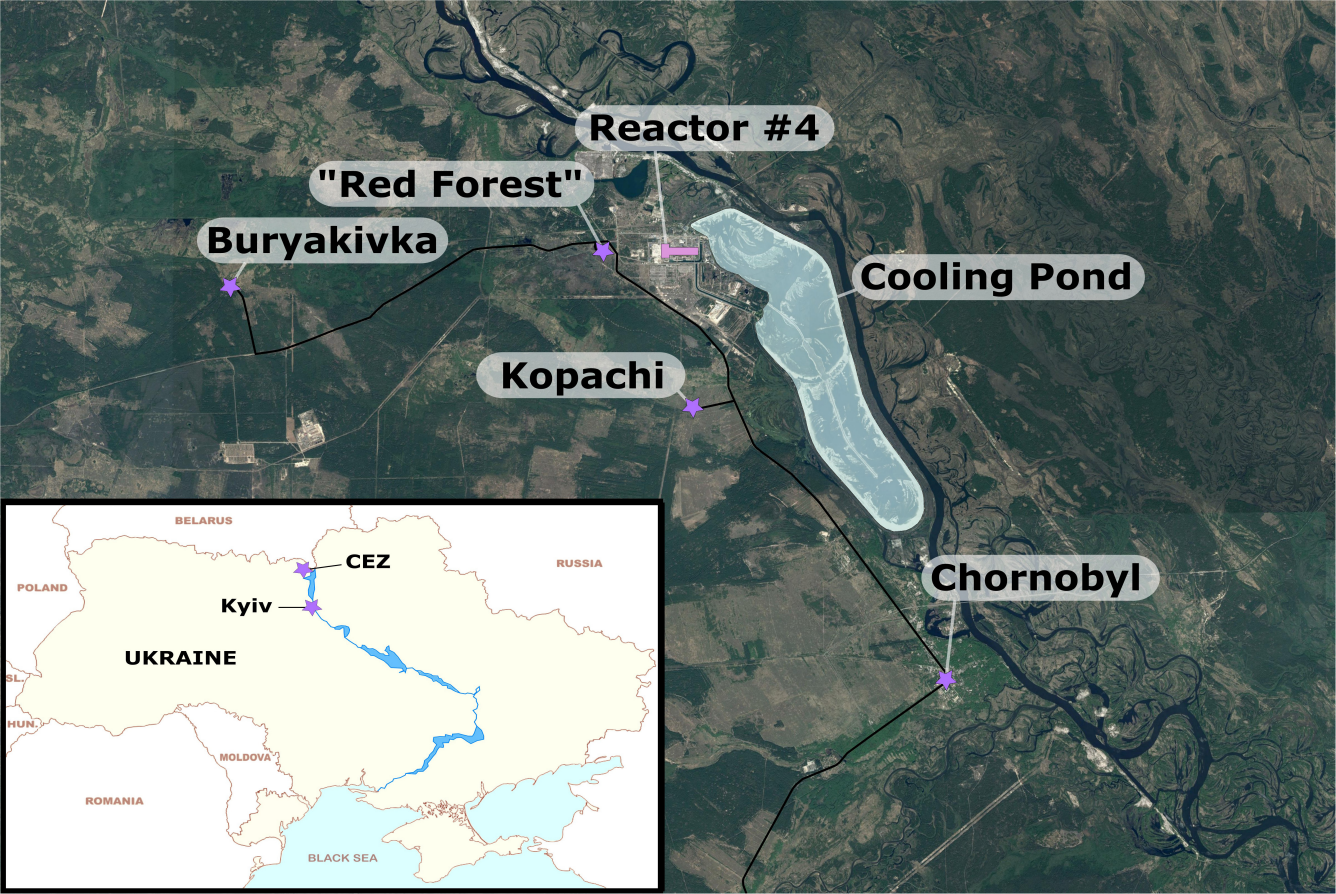
Study locations within the exclusion zone. Buryakivka and Kopachi are abandoned settlements and the “Red Forest” is a natural area.

One of the advantages of the fixed-wing is its increased range and speed relative to multi-rotor systems. Take-off and landing zones do not need to be close to the proposed survey area due to the higher flight speeds resulting in short transit times. Assuming the survey area was well known to the operators (or sufficiently well-characterized by pre-flight and ground surveying methods), it would be entirely possible to operate a few kilometers away from a survey area. Being able to operate from a distance is a key parameter in responding to nuclear incidents, as it allows operators to be completely or partially removed from a potential hazard. At present, current safety regulations regarding flying beyond visual line of sight (BVLOS) make this type of operation difficult to implement routinely. However, it is possible to obtain exemptions for such flights. It is envisaged that the response to a nuclear incident would constitute a valid exemption to this restriction under a well planned safety case. The work presented within this study presents a new design for a lightweight fixed-wing radiation mapping UAS to monitor terrestrial radiation within post-disaster environments using a modified data processing procedure based on the “Spectral Windows” method. The study aims to demonstrate the advantages of deploying these systems to map relatively large areas of contaminated land in a short time frame within the CEZ (Figure 1) and discusses their suitability to the task vs. the traditional methods of mapping radiation in the environment. It is hypothesized that the utilization of these systems can bridge the gap in spatial resolution, aerial coverage and operating costs that exist between previous UAS surveys and surveys conducted using manned aircraft.

## 2. Instruments and Methods

### 2.1. Fixed-Wing Mapping System

**Figure 2:**
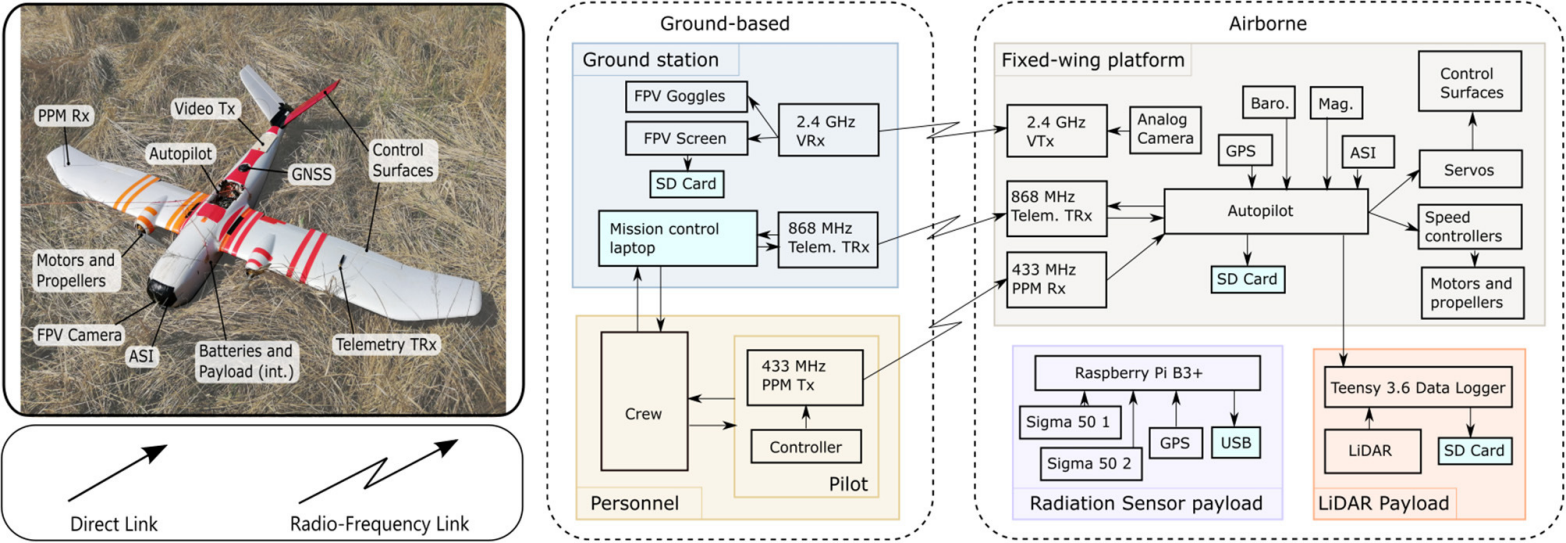
The fixed-wing vehicle and sub-system interconnection diagram.

The UAS used within this investigation comprises a fixed-wing vehicle, an integrated radiation mapping payload, and associated ground support equipment. The vehicle was custom built at the University of Bristol based around the “Titan” airframe (Skywalker, Shenzhen, China) (Figure 2). The aircraft has a wingspan of 2.1 m and a take-off weight of 8.5 kg (1 kg payload). This particular system was advantageous because it could be hand-launched and recovered by parachute, therefore allowing deployment at any site with a reasonable clearing without a requirement for a runway. Power was provided by a 12.7 Ah, 6S 22.2 Lithium Polymer (LiPo) battery, giving an approximate flight duration of 50–70 min depending on the weather conditions during the flight.

The vehicle featured a full auto-pilot computer with supporting sensors [GNSS, barometric altitude, airspeed indicator (ASI), and IMU]. The autopilot was capable of navigating the aircraft along pre-planned waypoint missions. Three wireless links were used to interact with the vehicle during flight. The pilot safety link, operating on the 433 MHz frequency, was used for initializing the automatic flight and for manual control during the parachute landing. The second link was a bi-directional telemetry modem operating on the 868 MHz frequency and was used for monitoring of flight statistics (such as battery consumption) and to issue updated commands to the autopilot. The third link was a live FPV video stream from a camera in the nose of the aircraft operating on the 2.4 GHz frequency.

There are three internal cabins within the body of the “Titan”. The first is the fuselage, which housed the control systems and batteries. The second is the payload bay, which contained the radiation sensor and its associated electronic control systems. The final cabin, located toward the tail of the aircraft, contained the parachute landing system and video transmitter.

The radiation sensing payload, supplied by Imitec (Bristol, UK), followed a similar design to the detection systems utilized within previous works by the authors (Martin et al., 2016, 2018; Connor et al., 2018a,b). However, modifications were made to the system design in anticipation of the new challenges posed by operating on a fixed-wing aircraft over a multi-rotor UAS. Whilst the previous system comprised of one solid state radiation detector, the updated system utilizes two SIGMA-50 CsI(Tl) scintillation detectors (Kromek Ltd., County Durham). This was implemented to increase the active detection volume of the system in order to offset the reduction in measured count-rates associated with operating at an increased altitude. An independent GNSS sensor allowed the radiation sensor payload to operate independently of the flying hardware with the exception of 12 V power supply. A secondary payload, comprising a higher power laser-range finder, was added by UOB to increase the fidelity of the height above ground measurements required for post-processing the radiation data. It must be noted that the data collected by the laser-range finder unit was not utilized within the processing in this study. This was due to practical considerations relating to the variable vegetation cover experienced across the surveyed area. The data from this system would have been used in an environment wherein vegetation cover does not extremely distort the apparent form of the land surface (e.g., an arid or urban environment).

### 2.2. Survey Locations

**Figure 3:**
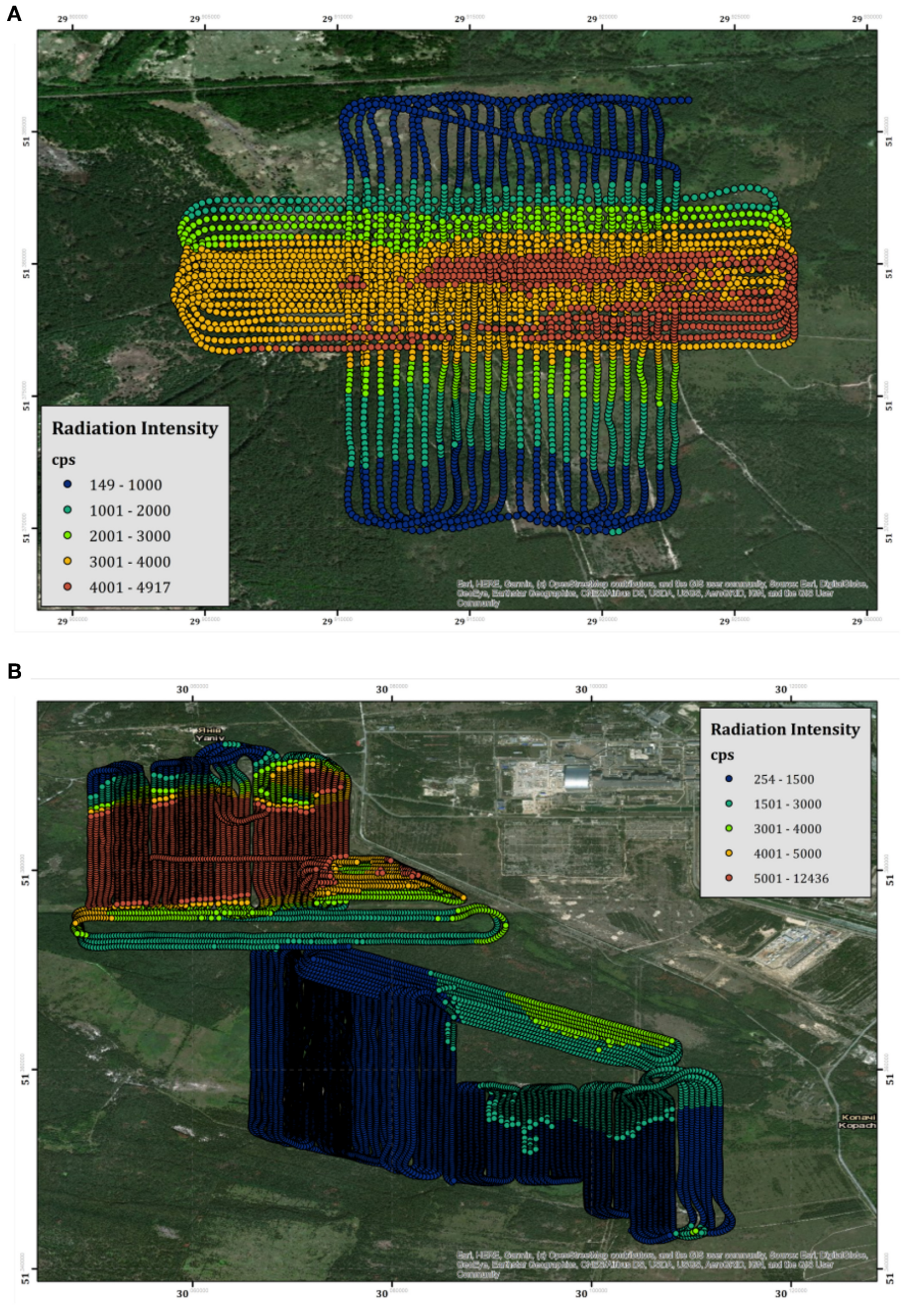
A map showing all the measurements recorded by the fixed-wing system within **(A)** the Buryakivka testing region and **(B)** the region between the “Red Forest” and Kopachi. The points are color-scaled according to their unprocessed total count intensity.

There are two separate zones in which the fixed-wing system was deployed within the CEZ. The first region lies between 12 and 13 km due west of the ChNPP [51.379 *N*, 29.916 *E*] (Figure 3A), near the former village of Buryakivka. This survey area covers 2.4 km^2^ and bisects the main westward trending plume of radioactivity deposited from the accident. This area was used as an accessible initial test site to ensure the system behaved as expected within a lower-risk, but real-world, environment. The second region was significantly larger than the first, covering 12 km^2^ of the land immediately to the west and south of the ChNPP Reactor 4 location. This area encompasses the “Red Forest” of Chornobyl [51.385 *N*, 30.051 *E*], as well as extending as far south as a former mechanical/farm yard near the settlement of Kopachi [51.345077 *N*, 30.111382 *E*] (Figure 3B).

### 2.3. Survey Methodology

#### 2.3.1. Operational Restrictions

Operating UASs in real-world environments is subject to nationally varying sets of restrictions. These restrictions are in place to maintain airspace safety for all aerial users. Within the CEZ, all flights were conducted under standard visual line-of-sight (VLOS), meaning that direct and unaided visual contact with the aircraft was required at all times. It is not sufficient to be able to just physically see the aircraft within VLOS restrictions, instead the pilot must be able to ascertain the orientation of the vehicle and be able to avoid other air users and/or obstacles should they be present within the airspace. A number of take-off and land zones were therefore used to split the CEZ into several zones that could maximize coverage within the flight restrictions. No extra modifications were required to operate under these conditions. A minimum separation distance of 1 km from the New Safe Confinement Building (the old Reactor Four site) was also imposed in addition to the standard restrictions.

#### 2.3.2. Flight Planning and Deployment

Each of the flights conducted using the fixed-wing system in the CEZ followed the same operational process. Once a target region was chosen, a small multi-rotor UAS (DJI Phantom 4 Pro) was deployed to investigate the area for obstacles and potential radio interference sources to determine the minimum altitude that the fixed-wing system can safely operate. The minimum altitude was set by adding a margin of 15 m to the measured altitude of the tallest structure present. Wherever possible, operating as low as reasonably practical was the target for every survey. Minor variations in the vegetation canopy height and the land surface throughout the zone resulted in survey altitudes of between 40 and 60 m above ground level (agl). Flight velocities were programmed to operate between 14 and 18 m s^−1^ airspeed, although local wind conditions created significant variations in the resulting ground-speed.

Once the flight altitude was decided, the flight paths were planned in a parallel-line raster scan format. The separation distance between each of the flight lines was equal to the survey altitude in all cases. At this flight line separation, the half-angle of the detectors produce a circle of investigation with a radius equal to the height above ground level of the aircraft (Martin, 2019, p. 77). Therefore, in the current flight planning configuration, the detector system is considerably oversampling the land; the measurement footprint from one flight line ends along the ground trace of the previous flight line. Whilst this is counter-intuitive to aiming to maximize spatial coverage, this compromise was necessary to demonstrate the range of the system whilst still maintaining VLOS with the aircraft. As the fixed-wing system has an inherent turning radius that is larger than the flight line separation, the parallel grid pattern was produced by flying in a series of laterally-offset loops rather than by flying each flight line in turn from one end of the survey to the other.

The surveys within the CEZ were completed semi-autonomously. The aircraft was hand-launched in automatic flight mode on take-off and remained in automatic mode until the landing phase of the flight. During the main flight phase, internal parameters (battery voltage, airspeed, ground-speed etc.) were monitored by the co-pilot using the ground station software. Whenever necessary, certain parameters were altered in-flight by the co-pilot under command from the pilot. Only the landing phase of the flight was conducted manually, with the pilot taking control of the aircraft upon its return to the take-off location. The automatic return to home function was programmed to bring the aircraft directly back to the take-off location whilst climbing to 70 m agl. Due to the lack of appropriate landing strips within the CEZ, the physical landing of the aircraft was accomplished through the deployment of a parachute.

### 2.4. Ground Investigation of Aerial Data

To further investigate the radiological measurements collected by the UAS, a series of ground-based measurements were collected within areas that were accessible to the ground team over the 6 days of data. These measurements were collected using a series of PED+ personal dosimeters (from Tracerco^TM^) and ground-based versions of the fixed-wing mapping system presented in this study. The dosimeters were placed on the body of each of the team of six operators within the CEZ and continuously collected georeferenced dose-rate measurements every 2 s, allowing for aerial measurements to be correlated against ground measurements. The ground-based mapping systems were deployed as handheld devices that were carried by the operators throughout the target areas. Wherever safe and permissible, measurements were recorded by walking in a series of parallel grid lines to ensure optimal coverage throughout the area. More direct routes and quantification pathways were used if the dose-rates were considered high in order to minimize exposure to operators.

### 2.5. Calibrations and Data Processing

Using aerial platforms to collect measurements about the Earth's surface often requires a number of processing steps to correct the raw data to more appropriately reflect the original signal. This is because the collected signal may go through some elements of change during its travel between the source and the detector. The same is especially true for aerial gamma-ray measurements. The following procedure outlines the process used within this study to convert the raw gamma-ray spectra obtained by the fixed-wing system into a cesium equivalent dose-rate (CED), in μ*Sv h*^−1^, at 1 m agl.

The raw 10 Hz measurements recorded by the system were first integrated into 1 Hz intervals and corrected for the dead time of the detector during the measurement. Then a correction for the cosmic, aircraft and radon background signal was applied to the data. This correction factor was determined by hovering the detection system at incrementally increasing altitudes over a wide meander bend of the River Pripyat, which is located <20 km from both the Buryakivka and Red Forest survey regions. The water within the river acts as an attenuating medium for the terrestrial signature of the total radiation flux within the surrounding area. With the terrestrial signal removed, the resultant recorded spectrum represents the contribution of all other sources of radiation within the environment. This derived background signal was subtracted from every terrestrial spectrum recorded within the CEZ.

To keep integration times as short as possible and maximize the achievable spatial resolution of the surveys, a fast Fourier transform (FFT) was applied to the background-corrected spectrum to remove high-frequency noise. The appropriate filtering magnitudes/masks for this task were determined experimentally by exposing the detection systems to a small source of ^137^Cs for intervals of a few seconds and visually inspecting the resultant unfiltered and filtered spectra. The energy range of the Sigma-50 detectors used within the payload is reported as 0 - 2 MeV. However the detection efficiency is significantly reduced beyond 1.2 MeV, meaning that observable signals within the potassium and uranium windows are unlikely even after FFT filtering. Hence, spectral stripping cannot be applied. Instead, a third-order polynomial estimation of the baseline of the region surrounding the ^137^Cs spectral window was calculated and subtracted from the FFT spectrum, removing the Compton background below the peak and leaving only the direct measurements of ^137^Cs.

**Figure 4:**
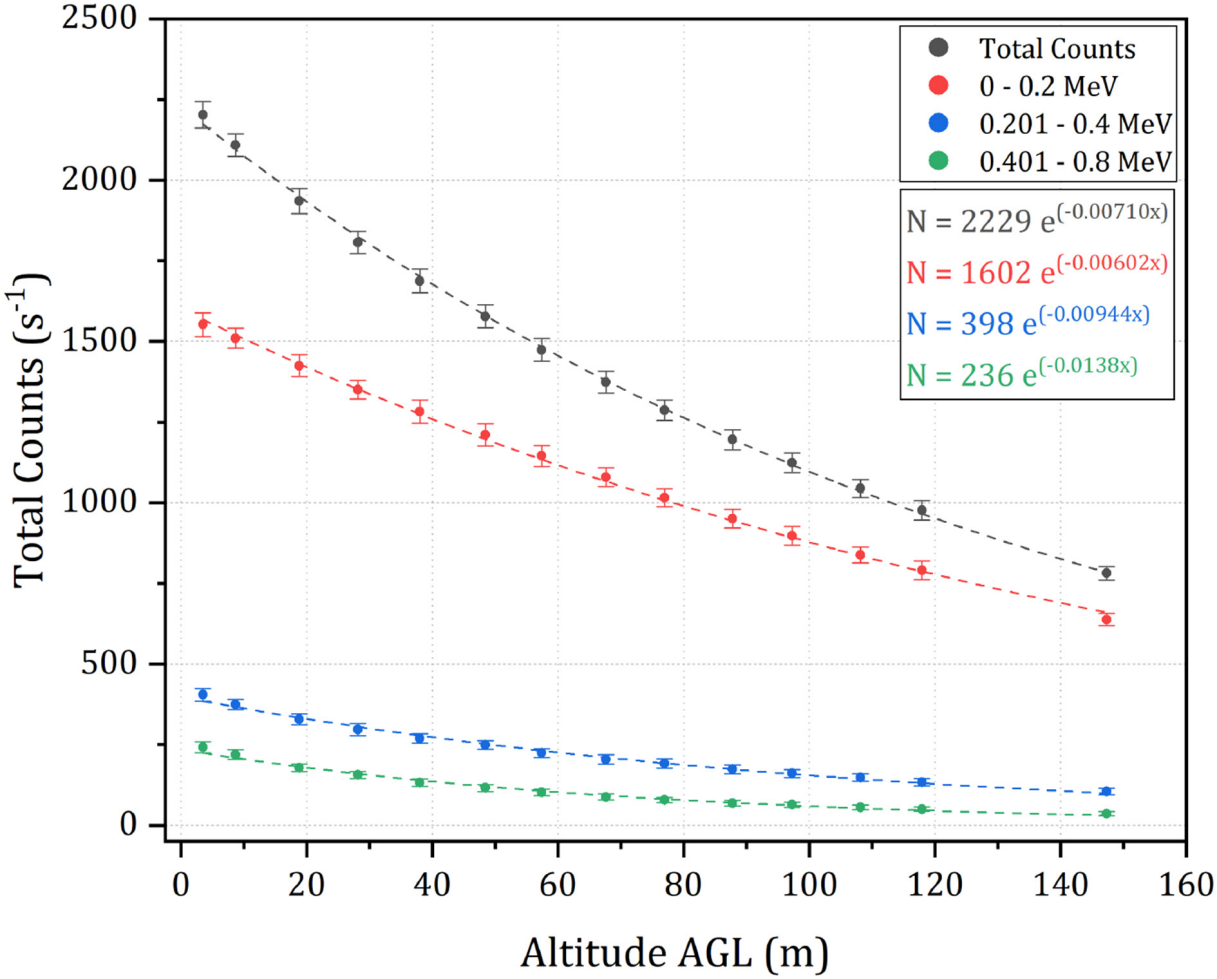
A scatter plot depicting the results of the hover survey used to determine the altitude correction curves for the CEZ for all four energy groupings. The error bars for each measurement are equal to one standard deviation of the data.

Whilst the aircraft operates at a consistent altitude above the take-off location, the ground surface underneath the aircraft can vary significantly throughout the planned route. As a result, the separation distance between the detector and its target is also varying, which erroneously creates high or low artifacts within the recorded data set. To overcome this issue, the measurements were corrected to a consistent one meter agl using a correction curve derived from hovering the detection system at incrementally increasing altitude overland in the CEZ. This was conducted using a multi-rotor UAS over a fixed-point within a area of land presenting a uniform radiological signature throughout the field of view (FOV) of the detector at the highest altitude within the survey. Spectral information was collected for 90 s at while hovering at each of the 14 altitudes across the range 3 - 150m agl. The resulting spectra were split into four groups, delineated by the energy of the recorded photons (total counts, 0–0.2, 0.2–0.4, and 0.4–0.8 MeV), and the relationship between the altitude and energy-specific intensity was determined by fitting an exponential regression line in the form of Equation (1) (Figure 4).

(1) $$\begin{array}{l} I=I_{0}.e^{-kx} \end{array}$$

In this relationship, *I* is the measured radiation intensity at altitude, *x*, agl, *I*_0_ is the intensity at ground level (*x* = 0) and *k* is an experimentally derived constant encompassing the contributions of geometric dilution and attenuation by the atmosphere between the detector and the source.

The height agl of the system was obtained by subtracting the land surface height, obtained from an SRTM 30 m digital elevation model (NASA JPL, 2013) of the CEZ, away from the GPS altitude of the UAS. For this application of converting measurements to dose-rate at 1 m agl, correcting directly to ground level using the DEM was preferred over using the single-point laser range-finder. This is because the pulsed laser signal produced by the unit can be intercepted by surfaces (tall vegetation canopies in particular) before reaching the target ground surface, which can produce false altitude artifacts/inconsistent correction surfaces within the data. In an environment presenting less variability from vegetation cover, the inbuilt laser range-finder would have been used over the DEM-based correction.

**Figure 5:**
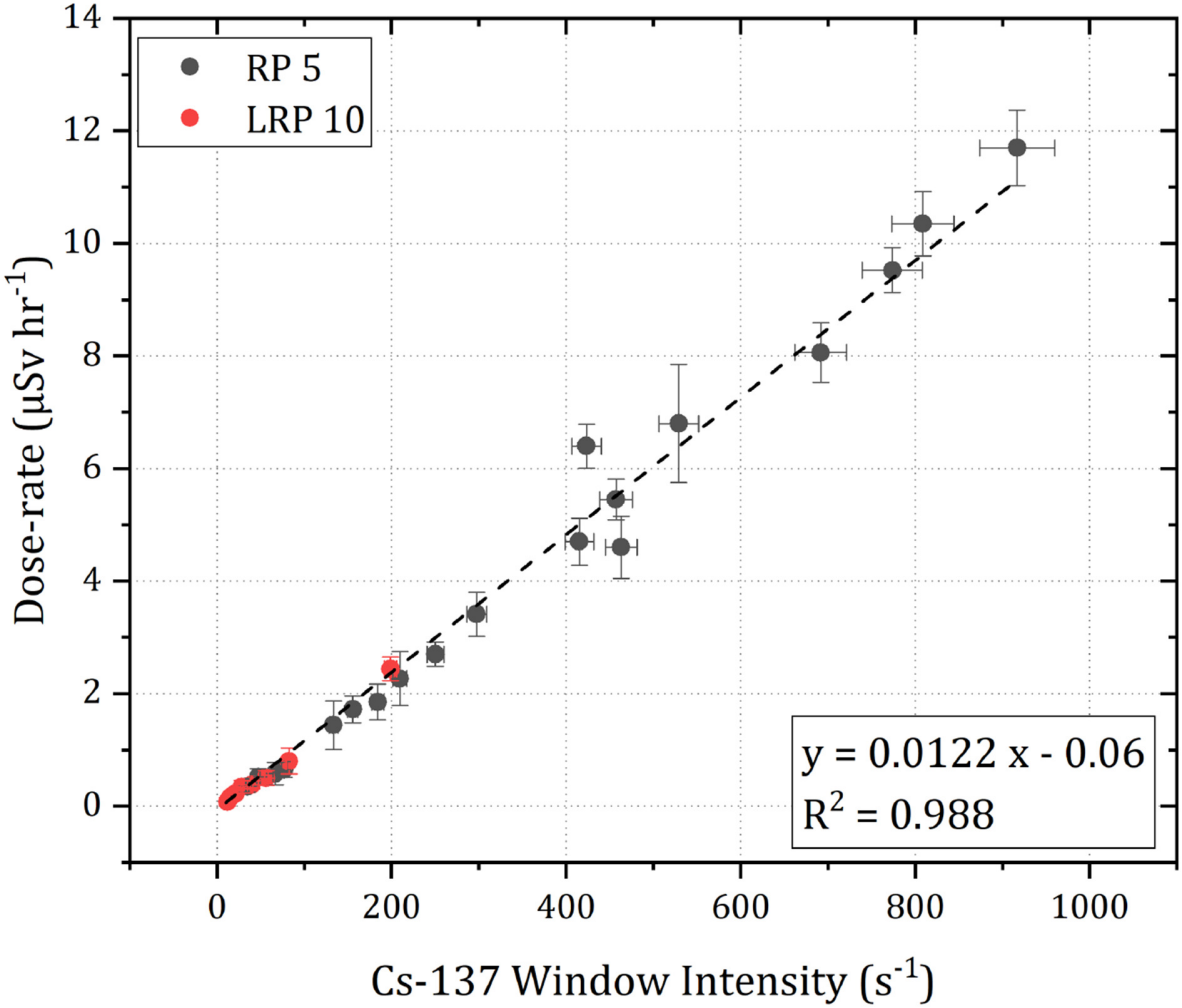
A scatter plot detailing the relationship between the net peak counts of ^137^Cs recorded by the detector and the dose-rate resulting from ^137^Cs from two sources of different intensities.

Following the altitude correction of each measurement within the survey, the intensity of ^137^Cs at 1 m agl are converted into a CED using a laboratory defined calibration. This conversion factor was determined by placing both the detection system and a PED+ personal dosimeter (from Tracerco™) at a range of distances away from several different sources of ^137^Cs. In total, two sources were used (labeled RP5 and LRP10), varying in activity between 500 and 1,500 kBq. Background measurements were recorded for both sensors for 30 min and normalized with respect to live time. For the active measurements, both sensors were exposed to the source for 5 min at the same separation distance. The time series data recorded by the dosimeter and the net peak area of the ^137^Cs peak were extracted and corrected for the background of the laboratory. The conversion factor between the CED and measured ^137^Cs intensity is represented by the line of best fit of a scatter plot of the two variables (Figure 5).

## 3. Results

### 3.1. Flight Characteristics and Duration

Over the 6 days of active fieldwork (17th April–24th April 2019), a total distance of 583.8 km was flown by the fixed-wing system, covering a total area of 14.8 km^2^. Table 2 shows the flight metrics for all flights conducted with the fixed-wing UAS. The system spent a total of 09h:17m:37s airborne, with an average flight time of 39m:50s. Buryakivka was the region in which the largest number of flights were conducted as this area was used as a semi-controlled testing zone. These test flights were used to fine tune all the aspects of the deployment procedure, including the pre-flight surveys, take-off and landing phases before operating closer to the ChNPP itself. The altitudes and flight velocities were varied slightly between each flight to reach the optimal level that the pilot was comfortable to operate subsequent surveys at. From these flights, it was determined that the optimal flight velocity was between 14 and 17 ms^−1^ airspeed, although in real-terms, the ground-speed value varied between individual flight paths within the same survey due to local wind conditions.

**Table 2:** Details of all of the flights conducted with the fixed-wing UAS within the CEZ.

**Flight code**	**Date**	**Altitude (m)**	**Flight Length (km)**	**Duration (min:s)**
Buryakivka Titan 1	17-04-2019	50	28.2	30:05
Buryakivka Titan 2	17-04-2019	45	28.0	31:51
Buryakivka Titan 3	17-04-2019	40	34.9	28:47
Buryakivka Titan 4	18-04-2019	40	42.7	39:02
Buryakivka Titan 5	18-04-2019	50	51.2	60:24
Buryakivka Titan 6	18-04-2019	50	51.7	48:42
Kopachi Titan 1	20-04-2019	50	52.9	52:23
Kopachi Titan 2	21-04-2019	50	60.1	65:58
Kopachi Titan 3	22-04-2019	50	39.7	30:11
Kopachi Titan 4	22-04-2019	50	39.1	30:36
Kopachi Titan 5	22-04-2019	60	39.8	30:31
RedForest Titan 1	23-04-2019	45	49.0	39:04
RedForest Titan 2	24-04-2019	45	44.6	39:23
RedForest Titan 3	24-04-2019	45	21.9	30:40

The largest average distance traveled in a single flight by the UAS was within the Kopachi region of the map [51.371 N, 30.065 E: 51.434 N, 30.114 E] with a value of 46.3 km. This region is dominated by open fields featuring small amounts of vegetation of <5 m in height, therefore presenting the most optimal conditions for all flight phases. The open fields provided space to take-off and land in any direction and permitted excellent visibility to the aircraft during the in-flight phase, allowing line-of-sight to be maintained easily. The longest distance covered was also in Kopachi, totalling 60.1 km in length. This value describes roughly the total distance that can be covered safely by the UAS given the current battery technology available to operators. If possible, all the flights used within the survey would be closer to this upper limit, but due to line-of-sight restrictions this was not possible in all parts of the surveyed areas.

### 3.2. Radiological Monitoring

#### 3.2.1. Buryakivka

**Figure 6:**
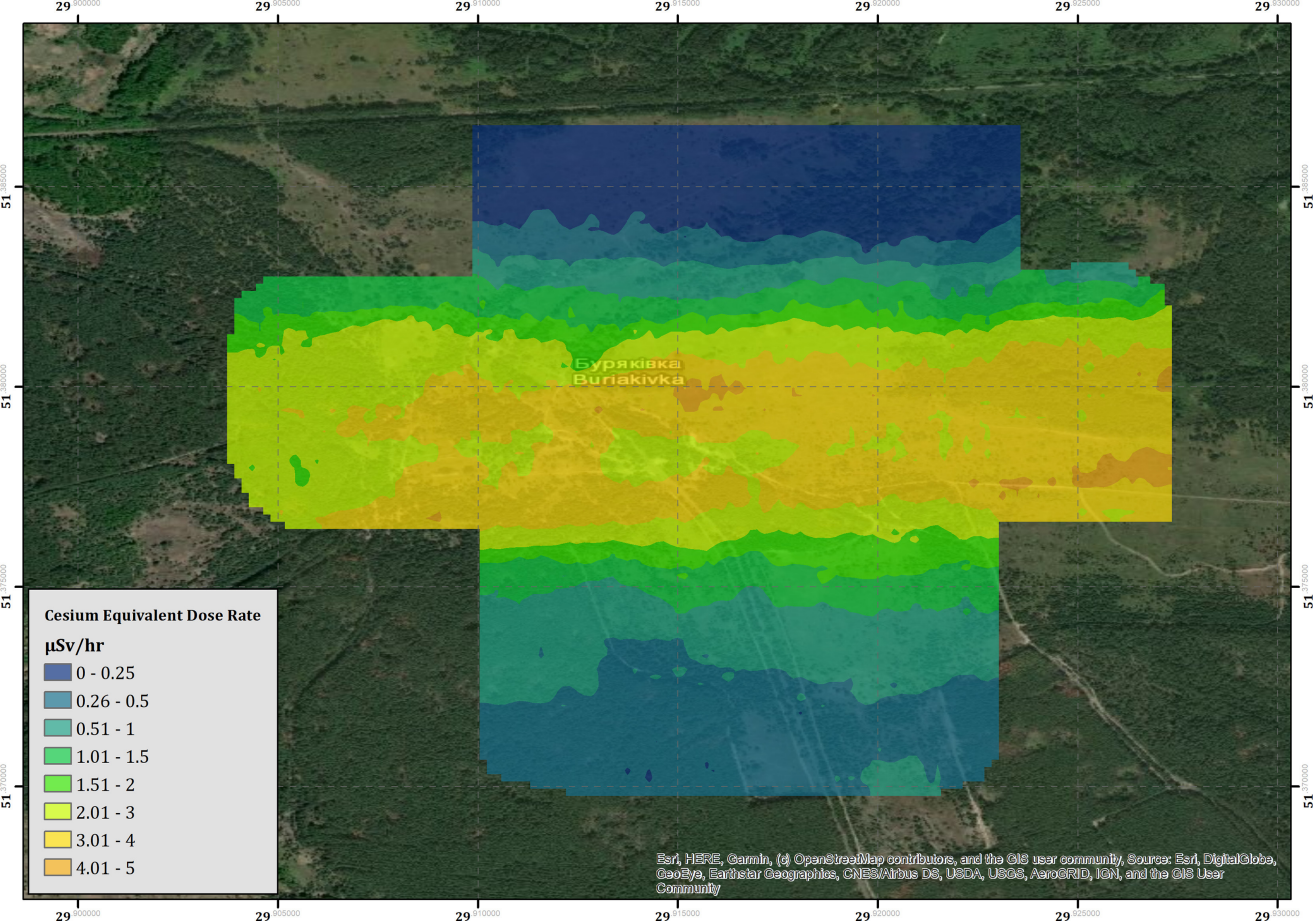
The cesium equivalent dose-rate (CED) of the Buryakivka area.

The results of the derived CED for the Buirakivka survey area are presented within Figure 6. The map within this figure is produced from three flights conducted at 40–45 m altitude agl, flights conducted at more elevated altitudes during the testing process have not been included within the map as many of these cover the same areas. An inverse distance weighting (IDW) interpolation algorithm has been applied to the data to produce the color-scaled CED overlay, which is presented at a pixel size of 20 × 20 m. This resolution was chosen as it is slightly coarser than the inline point spacing of the data set. The overall trend of the map follows the expected pattern from previous soil sampling investigations as presented within Kashparov et al. (2018), exhibiting a contaminant plume trending east to west, which drops off in intensity to the immediate north and south of the central line. The maximum CED measured within this area is 4.65 μSv h^−1^, measured at 51.363198 N, 30.107020 E, which is more than 23 times greater than the average total background dose-rate of the UK (0.2 μSv h^−1^).

#### 3.2.2. Red Forest and Kopachi

**Figure 7:**
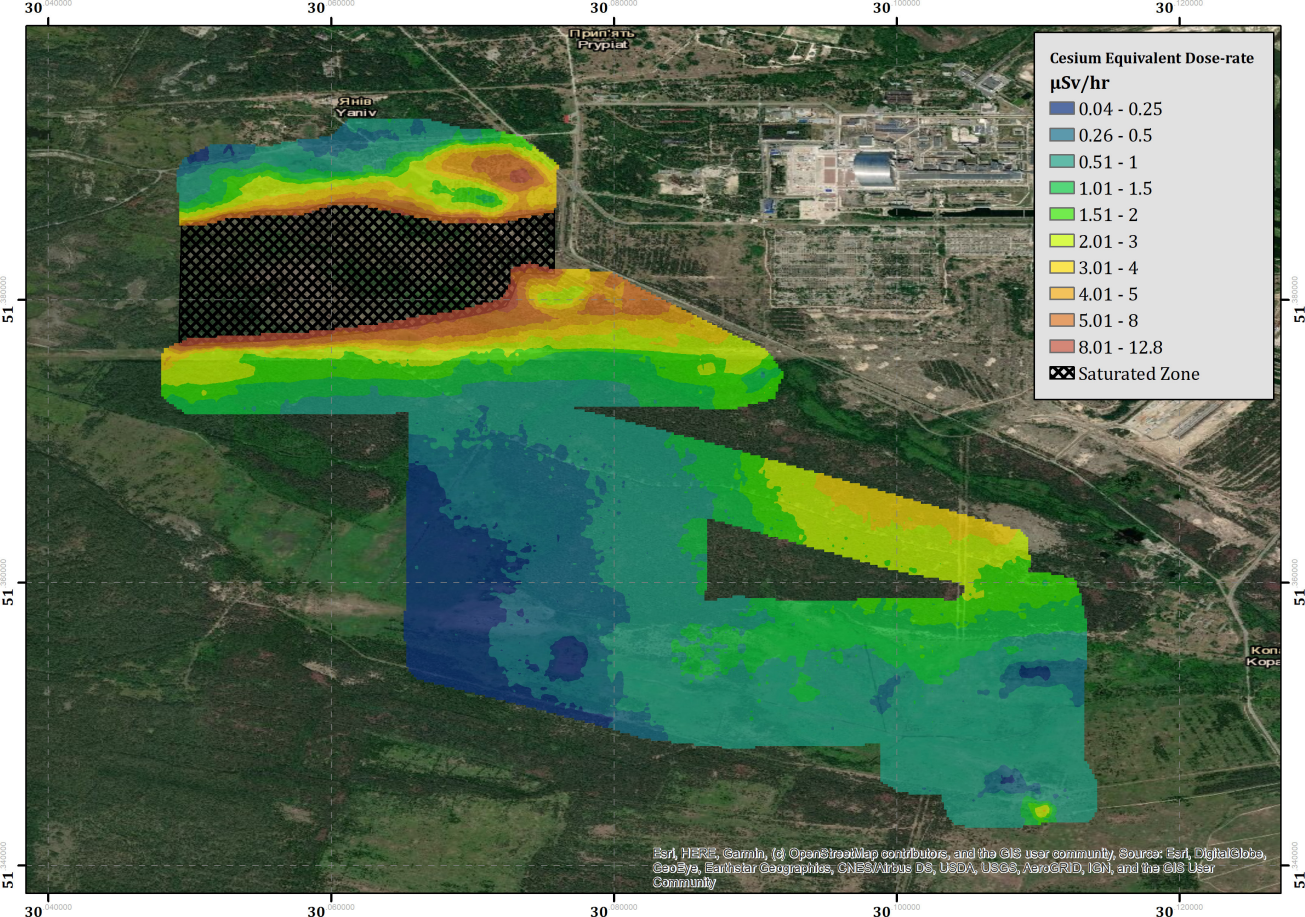
The cesium equivalent dose-rate (CED) of the “Red Forest” area surrounding the ChNPP.

The measured CED for the region surrounding the ChNPP is presented within Figure 7. The combined survey amalgamates the data from seven flights conducted over 4 days of deployment. Contrary to the data collected within the Buryakivka region (section 3.2.1), all the surveys conducted within this area are included within the presented data set (see Table 2 for full flight details). The color-scaled overlay is once more presented at a pixel size of 20 × 20 m. As expected, the overall CED measured in the area surrounding the ChNPP is significantly larger than that measured in Buryakivka. The maximum CED successfully recorded by the fixed-wing system was 12.8 μSv h^−1^, which is 2.8 times greater than the maximum CED recorded within the Buryakivka region. The map shows two main areas displaying elevated dose-rates. The first is a sharply delineated hot spot that extends immediately to the west of the ChNPP itself and covers the “Red Forest” area [51.379 N, 30.071 E]. The second is a much broader zone of elevated intensity, extending southwards from the plant toward the village of Kopachi [51.366 N, 30.100 E]. This overall trend is also depicted within the soil sampling investigations previously conducted by Kashparov et al. (2018), showing a general agreement between this dataset and previously published works from other institutions.

Located at the south-eastern corner of the area is a region of elevated dose-rate (3.3 μSv h^−1^) that lies within an area of relatively low dose-rate (0.51–1.0 μSv h^−1^). The hot spot [51.343843 N, 30.110399 E] manifests in an almost idealized point-source geometry when compared to the broad spreading of radioactivity evident within the measurements collected in the area surrounding it. The shape and location of the hot spot suggest that its presence is the result of anthropogenic concentration of radioactivity rather than the natural deposition following the accident. Dose-rate information could not be extracted from the cross-hatched area within Figure 7 due to detector saturation issues.

## 4. Discussion

### 4.1. Radiological Monitoring

Using the fixed-wing UAS, 15 km^2^ of the CEZ was successfully mapped, detailing the dose-rate variation relating to the ^137^Cs concentration in the ground and other surface features. Unfortunately, the high-intensity plume extending over the “Red Forest” could not be presented in its entirety as its significant radiological fingerprint caused an overloading (saturation) of the electronics of the detector. Whilst total count data could still be recorded, the measured gamma spectrum experienced significant degradation when exposed to total count rates of over 5,500 counts per second (cps). This reduction in signal quality manifests in the form of shifting the ^137^Cs peak toward a lower gamma energy (i.e., shifting the peak to the left) and significantly increasing the full width at half maximum (FWHM) value. This leads to non-sensical values within the analysis, which have been redacted from the map presented within this study. Detailed analysis of the spectral data indicates that reliable information can be extracted from the detector up to around 5,250 cps, as the peak positions and FWHM values remain within the manufacturers tolerance. Any measurements with a total count rate of greater than 5,250 cps have been removed from the presented dataset.

Previous surveys have measured dose-rates within a small portion of the “Red Forest” area to be up to around 170 μSv h^−1^ (Burtniak et al., 2018). These surveys were conducted within the portion of the “Red Forest” that could not be mapped by our system at much lower altitudes (5 m) and much slower velocities that are typical of multi-rotor surveys. Despite being inherently unreliable, the total-count data recorded by the fixed-wing system (Figure 3) reported a maximum count-rate of 12,436 cps at 45 m altitude. Even though the measurements were saturated, using this count-rate as a minimum value for the radiological intensity within this area would produce an expected dose rate of at least 95 μSv h^−1^ (based upon the approximate ratio of the altitude corrected total intensity to cesium dose-rate). As the detector is facing an overload during these measurements, the real total counts value would most likely be greater, producing a larger CED.

Overall, the general results from the UAS agrees well with previous datasets collected though other methods. The extensive soil sampling investigations published by Kashparov et al. (2018) provide excellent overall coverage and measurement accuracy throughout the CEZ, but fail to provide an easily repeatable method of monitoring radiation within post-disaster environments. The amount of labor-hours involved in conducting ground sampling surveys of this size are significant and the results are comparatively low-resolution when assessed against more mobile methods. The effects of this are best shown by the localized hot spot present near the south-eastern corner of the map [51.343843 N, 30.110399 E], which was previously unreported in literature until this study was conducted.

**Figure 8:**
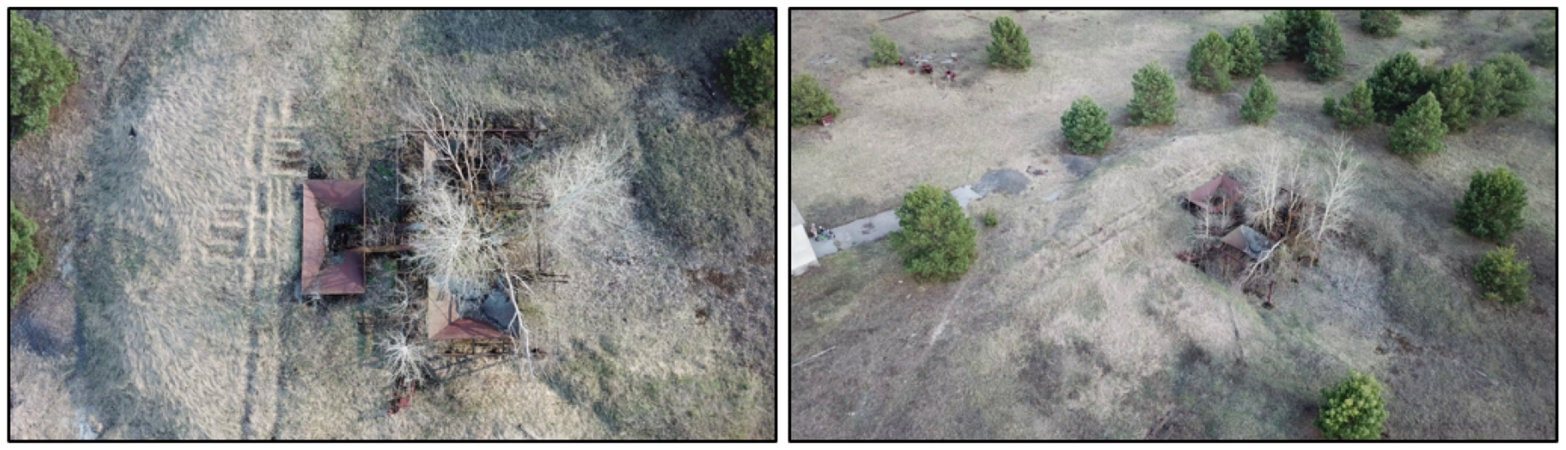
Aerial photographs of the “hopper” hot spot, presented in a plan view **(Left)** and a areal view in context of the local area **(Right)**.

Following the identification of the hot spot from the raw data collected by the aircraft, a ground-based team was deployed to investigate the area covered by the elevated intensity region. Upon arrival, this team used SIGMA-50 detectors, Geiger-Muller (GM) tubes and PED+ personal dosimeters to monitor the radioactive output of this region. The source of the radioactivity was determined to be a series of funnel-shaped metal structures that seem to have been used to mechanically sort through material in an attempt to reduce the overall volume of contaminated material following the accident (Figure 8). These structures will be referred to as “hoppers” for the remainder of this document. The residual radiological fingerprint of this process is significant. Ground-measurements, acquired using dosimeters, measured more than 2 mSv h^−1^ directly in the vicinity of the “hoppers.” Attempts at recording gamma spectrometry measurements were futile due to saturation issues.

The measurements collected by the aircraft at this point in space are significantly lower than the values measured by the ground team (3.3 μSv h^−1^ vs. 2 mSv h^−1^). There may be a number of reasons for the discrepancy between these values. Firstly, the analysis performed on the results collected by the aircraft focuses solely on the ^137^Cs signal, ignoring contributions from any other radionuclides (these are outside the scope of this study and will be investigated in future studies). The myriad of radioactive material released from the accident is highly complex and the measured contribution of ^137^Cs is but a component of the total output (Smith and Beresford, 2005; Burtniak et al., 2018). Given that the “hopper” hot spot is so intensely radioactive, the on-ground measurements could be recording inputs from other radionuclides in addition to the measured ^137^Cs signal. This could potentially include gamma-ray signals from ^241^Am, which emits a low energy gamma-ray (0.06 MeV) that is more easily attenuated by the medium between the source and the detector (see Figure 4). These kinds of signals are difficult to detect with any confidence at the altitudes used within this survey, especially because the incomplete transfer of energy between incoming photons and the detection crystal (very common for small-volume, room temperature detection systems Gilmore, 2008) creates a high background signal at the low energy range of the spectrum. Radionuclides other than ^241^Am and ^137^Cs are also expected to be present within the signal emanating from this region, including contributions from fission products from spent nuclear fuel.

Another reason for the observed disparity between the aerial measurements and the on-ground measurements is the short sampling time and FOV averaging at each point on the earth. As the system operates at a minimum velocity of 14 ms^−1^, the measured signal consists of a sampling area of 1,120 m^2^ for a single, 1-s measurement before the altitude correction is applied. Highly localized variations of a few meters in area will therefore not be fully resolved, instead being averaged with the surrounding area that constitutes a single measurement. This provides one possible explanation into the differences in expected dose-rate and measured dose-rate within this study. Once more, it must be noted that the dose-rate measured here considers only the dose-rate from ^137^Cs, whereas raw ground measurements recorded by personal dosimeters are collecting information from all sources (including natural radionuclides and other sources released during the accident). The time between the occurrence of the accident and this study is also an important consideration. In the three decades since the accident, radioactive material has had time to penetrate the ground surface and new sediment has had time to be deposited on top of the original radionuclide deposition. The burial of material means that there is not only more attenuating material between the detector and its target source, but the material is also much denser than air, resulting in fewer interactions with unscattered ^137^Cs photons with the active detection volume.

The overall mission objective to deploy the fixed-wing system within a real-world post-disaster environment has been achieved. As far as the authors are aware, this is the first time that this variation of a radiation mapping UAS has been deployed in a non-controlled situation to map terrestrial gamma radiation. Previous iterations of this detection unit for multi-rotor systems have been successfully utilized in similar environments within the Fukushima fallout zone in Japan, albeit these zones have been less intense than the levels of radioactivity experienced in the proximity of the “Red Forest.” One of the advantages of using lightweight UASs is that payloads can be altered, or completely removed, with ease. Given the observed limitations of the detectors used within the system at relatively high gamma fluxes, these would likely be changed in future iterations. Cerium Bromide (CeBr_3_) and Lanthanum Bromide (LaBr_3_) detection systems are being considered for future systems as these provide excellent energy resolutions and optical yields, even at small-volumes (Lowdon et al., 2019).

In terms of short-term improvements, one of the two Sigma-50 units will be swapped for a smaller-volume GR1 unit (a CZT semi-conductor detector from Kromek Group PLC, County Durham, UK). This detector has a better tolerance for high gamma fluxes and an improved energy resolution when compared to the Sigma-50 unit used within the current system. It is however, more vibrationally sensitive and will require some efforts to dampen these effects within the UAS payload. A further survey specifically aiming to map the saturated zone of the “Red Forest” (see Figure 7) using the updated system is planned for October 2019 to improve upon the results collected herein.

### 4.2. System Evaluation and Wider Applications

The results from the radiological investigations of the CEZ suggest that the fixed-wing system presented within this study is effective at mapping ^137^Cs distribution, although the significant radioactivity of the “Red Forest” proved to be too much for the detectors used within the payload. Whilst this conclusion satisfies the overall aim of this study, there are a few more considerations to be discussed before the system can be considered for use in more routine situations or be implemented into emergency procedures in the future.

UAS-based investigations are often at the mercy of the weather. Certain counter-measures can be implemented in some cases to overcome problems, for example, waterproofing the central electronic components can allow certain types of UASs to operate even in wet weather. However, the fixed-wing UAS used in this study is sensitive to variations in localized wind velocities. The surveys were conducted at a target velocity 14–18 ms^−1^, but on some occasions ground-speeds of up to 25 ms^−1^ were recorded during survey lines orientated such that the aircraft experienced a tailwind. As previously mentioned within section 2.3, the differences in wind velocities experienced by the UAS during individual legs of the same survey create inconsistencies in recorded data. The system records raw data at 10 Hz before being resampled in the post-processing phase into 1 Hz intervals. Differences in the velocity of the aircraft mean that the effective sampling area of each measurement varies throughout the survey. As a result, the pixel size has been increased slightly to a lower resolution in the final map to encompass some of this variation.

Even though UASs with similar or greater ranges have been reported within the literature, the fixed-wing UAS used within this study is considerably lighter than these reported platforms. The Yamaha RMAX platform utilized within Sanada and Torii (2014) weighs 100.5 kg with the under-mounted radiation mapping payload attached, whilst the system presented herein weighs 8.5 kg by comparison. Whilst the radiation mapping payload used within Sanada and Torii (2014) was able to carry a larger payload (resulting in larger detection volumes), the extra weight is significant in terms of the operation of UAS in the real-world. Regulatory restrictions exist for the operation of heavier platforms around the world as they present a greater hazard to the environment through the increased energy involved in an impact (Connor et al., 2016). As a result, it is easier to deploy lighter platforms within surveys globally.

The concept of utilizing fixed-wing vehicle for this task aims to bridge the gap between manned aircraft and multi-rotor capabilities. Currently, multi-rotor UASs represent the high-resolution end-member of the airborne radiation mapping spectrum, often achieving sub-10 m pixel sizes (Martin et al., 2015, 2016; Burtniak et al., 2018; Connor et al., 2018a,b). Manned aircraft systems (MASs) represent the opposite end of the scale, operating at between 90 and 200 m agl and achieving spatial resolutions of 200–500 m (Pitkin and Duval, 1980; Sanderson and Cresswell, 2008). Within this survey, operating altitudes were maintained at 40–60 m agl, with a spatial resolution of 20 m after the post-processing procedure. This successfully provides a middle ground between the two end-members, both in terms of the resolution and total coverage capabilities of the system. It is worth noting that the flight line separation can be increased if the survey values absolute coverage over spatial resolution. As the detector FOV increases linearly with altitude, the flight line spacing could be increased up to two times the altitude of flight without incurring a loss of net spatial coverage.

As well as bridging the gap between current methods, there is also the potential that manned aircraft could be superseded by using fixed-wing UASs in the future, especially with sufficient improvements in battery technology. This is especially true when considering financial factors. The total cost of building and deploying the UAS used herein was $24,000 (including all parts, labor costs for build and deployment and insurance costs), whereas a manned survey would be considerably more expensive. The cost for repeat surveys following the initial investment totals at $9,000 for the equivalent survey conducted within this study. This is based on salary estimates and operational costs for a three man crew over 6 days of active operation. If the equipment is used multiple times, the cost-benefit of the system is significantly improved over utilizing manned aircraft.

Without much prior familiarity of operating within the CEZ, the fixed-wing system was successfully deployed at as low an altitude as reasonably possible using information obtained from on the fly pre-flight surveys. With a good knowledge of the survey area, it would be possible to achieve much more. Overall, the authors believe that there is extreme promise in widely utilizing these systems for a number of survey applications in the future after the implementation of the improvements suggested herein.

## 5. Conclusions and Future Work

This study presents the most comprehensive radiation map of the CEZ ever produced from a UAS. Over the 6 days of active fieldwork with the fixed-wing system, 15 km^2^ was investigated in a high spatial resolution (20 m pixel^−1^). In total, more than 580 km were flown across the region in a total flight time of 09h:17m:37s. The system demonstrated that radiation mapping investigations using UASs can be launched from safe-zones outside contaminated regions and operated continuously for more than an hour before returning to the safe-zone to land. Some previous systems presented within the literature have been required to launch within the contaminated zones at the risk of the operators.

Due to issues with detector saturation, the area in the most radiologically intense portions of the “Red Forest” were not presented with the mapped CED as this information could not be reliably extracted from the spectra recorded over this area. This problem is hoped to be solved in the future by operating using a different dual detector set-up (Sigma-50 and GR1 combination). In this configuration, the Sigma-50 would be used to map the areas displaying lower contamination concentrations (as presented by more than 85% of the area mapped in this study) and the GR1 would be used to map the areas wherein the Sigma-50 was saturated.

One of the most interesting findings was the presence of the previously unreported, anthropogenically-enhanced hot spot located in the south-eastern corner of the surveyed area. With the knowledge that the 2 mSv h^−1^ hot spot exists, a coordinated ground sampling investigation will be conducted to determine the nature of the radionuclide content and correlated against the measurements collected by the aerial platform.

The work conducted within the CEZ was part of a multi-faceted field investigation using numerous types of radiation monitoring methods. These included both fixed-wing and low-altitude multi-rotor UAS surveys, as well as ground-based monitoring methods using both tracked robots and humans. The data presented herein will be combined with the measurements recorded using the other methods in future works to complete a comprehensive radiological survey of the CEZ using mobile radiation monitoring methods. The demonstration of this system in this environment has further-reaching consequences than just the monitoring of post-disaster environments. With alterations to the included detection systems, using recommendations from Lowdon et al. (2019), this system could become a low-cost solution to monitoring large areas of land for mineral resources. This could be of particular interest to developing countries who currently struggle to conduct mineral reserve estimates due to the high expenditure involved in chartering manned-aircraft surveys. Further work within the CEZ is planned for October 2019 and April 2020.

## Data Availability Statement

The datasets generated for this study are available on request to the corresponding author.

## Author Contributions

Data collection was conducted by DC, KW, PM, YV, SG, IC, and TS. Continual aircraft development was conducted by KW and TR, with the piloting of the aircraft performed by KW. Data processing was conducted by DC with assistance from KW and DM-S, with necessary local information provided by IC and SK. Original manuscript preparation was conducted by DC. Further manuscript developments and improvements were performed by DC, KW, PM, TS, DM-S, SG, IC, TR, SK, and NS.

### Conflict of Interest

The authors declare that the research was conducted in the absence of any commercial or financial relationships that could be construed as a potential conflict of interest.
