# Corrigendum: Radiological Mapping of Post-Disaster Nuclear Environments Using Fixed-Wing Unmanned Aerial Systems: A Study From Chornobyl

**DOI:** 10.3389/frobt.2020.00030

**Published:** 2020-02-28

**Authors:** Dean T. Connor, Kieran Wood, Peter G. Martin, Sevda Goren, David Megson-Smith, Yannick Verbelen, Igor Chyzhevskyi, Serhii Kirieiev, Nick T. Smith, Tom Richardson, Thomas B. Scott

**Affiliations:** ^1^Interface Analysis Centre, University of Bristol, Bristol, United Kingdom; ^2^Aerospace Engineering, University of Bristol, Bristol, United Kingdom; ^3^South West Nuclear Hub, University of Bristol, Bristol, United Kingdom; ^4^SSE Ecocentre, Ministry of Ecology, Chornobyl, Ukraine; ^5^National Nuclear Laboratory, Workington, United Kingdom

**Keywords:** radiation, Chornobyl, UAS (unmanned aircraft system), fixed-wing aerial surveys, post-disaster, cesium, nuclear, drones (UAV)

In the original article, there was a mistake in Figure 6 and Figure 7 as published. The published dose-rates are incorrect due to a typing error within the code used to process the radiation data. Instead of correcting the measurements for the live time of the detector (TL), the typing error caused the intensity measurements to be adjusted according to the uncorrected sample time (TR). This affects the dose-rate magnitude of all the measurements presented but does not affect the trend or the reliability of the dataset. The corrected Figure 6 and Figure 7 appears below.

**Figure 6:**
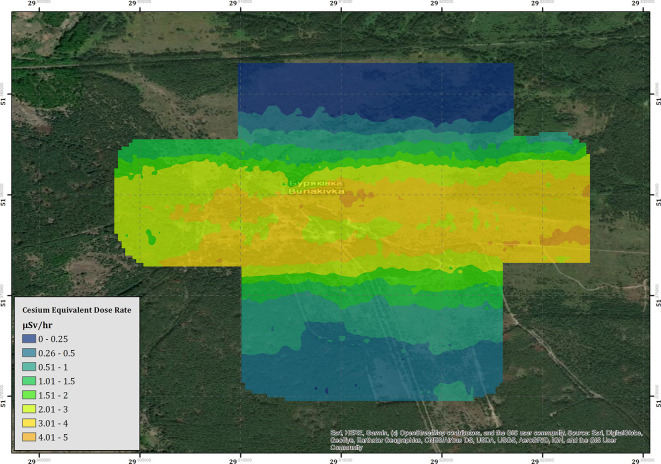
The cesium equivalent dose-rate (CED) of the Buryakivka area.

**Figure 7:**
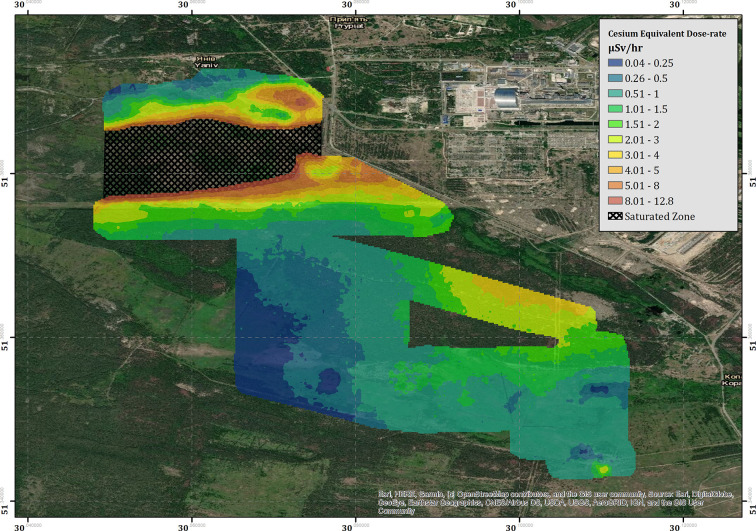
The cesium equivalent dose-rate (CED) of the “Red Forest” area surrounding the ChNPP.

A correction has been made to Section 3.2 (Radiological Monitoring), Section 3.2.1 (Buryakivka), Paragraph 1:

“The results of the derived CED for the Buirakivka survey area are presented within Figure 6. The map within this figure is produced from three flights conducted at 40–45 m altitude agl, flights conducted at more elevated altitudes during the testing process have not been included within the map as many of these cover the same areas. An inverse distance weighting (IDW) interpolation algorithm has been applied to the data to produce the color-scaled CED overlay, which is presented at a pixel size of 20 × 20 m. This resolution was chosen as it is slightly coarser than the inline point spacing of the data set. The overall trend of the map follows the expected pattern from previous soil sampling investigations as presented within Kashparov et al. (2018), exhibiting a contaminant plume trending east to west, which drops off in intensity to the immediate north and south of the central line. The maximum CED measured within this area is 4.65 μSv h^−1^, measured at 51.363198 N, 30.107020 E, which is more than 23 times greater than the average total background dose-rate of the UK (0.2 μSv h^−1^).”

A correction has also been made to Section 3.2 Radiological Monitoring, Section 3.2.2 Red Forest and Kopachi, Paragraphs 1 and 2:

“The measured CED for the region surrounding the ChNPP is presented within Figure 7. The combined survey amalgamates the data from seven flights conducted over 4 days of deployment. Contrary to the data collected within the Buryakivka region (section 3.2.1), all the surveys conducted within this area are included within the presented data set (see Table 2 for full flight details). The color-scaled overlay is once more presented at a pixel size of 20 × 20 m. As expected, the overall CED measured in the area surrounding the ChNPP is significantly larger than that measured in Buryakivka. The maximum CED successfully recorded by the fixed-wing system was 12.8 μSv h^−1^, which is 2.8 times greater than the maximum CED recorded within the Buryakivka region. The map shows two main areas displaying elevated dose-rates. The first is a sharply delineated hot spot that extends immediately to the west of the ChNPP itself and covers the “Red Forest” area [51.379 N, 30.071 E]. The second is a much broader zone of elevated intensity, extending southwards from the plant toward the village of Kopachi [51.366 N, 30.100 E]. This overall trend is also depicted within the soil sampling investigations previously conducted by Kashparov et al. (2018), showing a general agreement between this dataset and previously published works from other institutions.”

“Located at the south-eastern corner of the area is a region of elevated dose-rate (3.3 μSv h^−1^) that lies within an area of relatively low dose-rate (0.51–1.0 μSv h^−1^). The hot spot [51.343843 N, 30.110399 E] manifests in an almost idealized point-source geometry when compared to the broad spreading of radioactivity evident within the measurements collected in the area surrounding it. The shape and location of the hot spot suggest that its presence is the result of anthropogenic concentration of radioactivity rather than the natural deposition following the accident. Dose-rate information could not be extracted from the cross-hatched area within Figure 7 due to detector saturation issues.”

A correction has also been made to Section 4.1 Radiological Monitoring, Paragraphs 2 and 5:

“Previous surveys have measured dose-rates within a small portion of the “Red Forest” area to be up to around 170 μSv h^−1^ (Burtniak et al., 2018). These surveys were conducted within the portion of the “Red Forest” that could not be mapped by our system at much lower altitudes (5 m) and much slower velocities that are typical of multi-rotor surveys. Despite being inherently unreliable, the total-count data recorded by the fixed-wing system (Figure 3) reported a maximum count-rate of 12,436 cps at 45 m altitude. Even though the measurements were saturated, using this count-rate as a minimum value for the radiological intensity within this area would produce an expected dose rate of at least 95 μSv h^−1^ (based upon the approximate ratio of the altitude corrected total intensity to cesium dose-rate). As the detector is facing an overload during these measurements, the real total counts value would most likely be greater, producing a larger CED.”

“The measurements collected by the aircraft at this point in space are significantly lower than the values measured by the ground team (3.3 μSv h^−1^ vs. 2 mSv h^−1^). There may be a number of reasons for the discrepancy between these values. Firstly, the analysis performed on the results collected by the aircraft focuses solely on the ^137^Cs signal, ignoring contributions from any other radionuclides (these are outside the scope of this study and will be investigated in future studies). The myriad of radioactive material released from the accident is highly complex and the measured contribution of ^137^Cs is but a component of the total output (Smith and Beresford, 2005; Burtniak et al., 2018). Given that the “hopper” hot spot is so intensely radioactive, the on-ground measurements could be recording inputs from other radionuclides in addition to the measured ^137^Cs signal. This could potentially include gamma-ray signals from ^241^Am, which emits a low energy gamma-ray (0.06 MeV) that is more easily attenuated by the medium between the source and the detector (see Figure 4). These kinds of signals are difficult to detect with any confidence at the altitudes used within this survey, especially because the incomplete transfer of energy between incoming photons and the detection crystal (very common for small-volume, room temperature detection systems Gilmore, 2008) creates a high background signal at the low energy range of the spectrum. Radionuclides other than ^241^Am and ^137^Cs are also expected to be present within the signal emanating from this region, including contributions from fission products from spent nuclear fuel.”

The authors apologize for these errors and state that they do not change the scientific conclusions of the article in any way. The original article has been updated.
